# Lab on a Biomembrane: Rapid prototyping and manipulation of 2D fluidic lipid bilayers circuits

**DOI:** 10.1038/srep02743

**Published:** 2013-09-25

**Authors:** Alar Ainla, Irep Gözen, Bodil Hakonen, Aldo Jesorka

**Affiliations:** 1 Department of Chemical and Biological Engineering, Chalmers University of Technology, Kemivägen 10, 41296 Göteborg, Sweden; 2 Current address: Bio-Acoustic-MEMS in Medicine (BAMM) Laboratory, Center for Bioengineering, Department of Medicine, Brigham and Women′s Hospital, Harvard Medical School, Boston, MA, USA.

## Abstract

Lipid bilayer membranes are among the most ubiquitous structures in the living world, with intricate structural features and a multitude of biological functions. It is attractive to recreate these structures in the laboratory, as this allows mimicking and studying the properties of biomembranes and their constituents, and to specifically exploit the intrinsic two-dimensional fluidity. Even though diverse strategies for membrane fabrication have been reported, the development of related applications and technologies has been hindered by the unavailability of both versatile and simple methods. Here we report a rapid prototyping technology for two-dimensional fluidic devices, based on in-situ generated circuits of phospholipid films. In this “lab on a molecularly thin membrane”, various chemical and physical operations, such as writing, erasing, functionalization, and molecular transport, can be applied to user-defined regions of a membrane circuit. This concept is an enabling technology for research on molecular membranes and their technological use.

Supported molecular phospholipid films are versatile model membrane architectures^1^, which are valuable to mimic fundamental properties and features of the plasma membrane at reduced complexity^2^,^3^,^4^. Double bilayer^5^,^6^, single bilayer^7^,^8^,^9^,^10^,^11^,^12^ as well as monolayer films^13^ can be formed on solid supports, providing enhanced stability and improved accessibility by probing techniques^14^. Supported membranes can cover an extensive area homogenously, which greatly facilitates modification, observation and imaging^1^. Two-dimensionality and fluidity allow their utilization in micro-15,16,17,18 and nanofluidic devices^19^, which supports functional studies of membrane proteins^20^,^21^, and promotes the development of membrane-based chemistry^22^, sensing^23^,^24^ and separation^15^. Here we introduce a microfluidic toolbox to write 2D nanofluidic networks composed of supported phospholipid membranes, and dynamically modify their connectivity, composition, and local function. We demonstrate how such networks are conveniently generated and locally restructured, and show how various design possibilities such as diffusional barriers and hydrodynamic trapping points can be used in a “lab on a biomembrane” to directly address biomembrane functions and properties, or to perform membrane-assisted studies of molecular interactions. Our open volume approach is fundamentally different from the miniaturized technologies currently used to assemble artificial bilayer systems^18^. Microfluidic devices that operate in the “open space”, i.e., outside the confinement imposed by channels and chambers, provide unique opportunities for interacting with biological samples.

## Results

**Figure 1:**
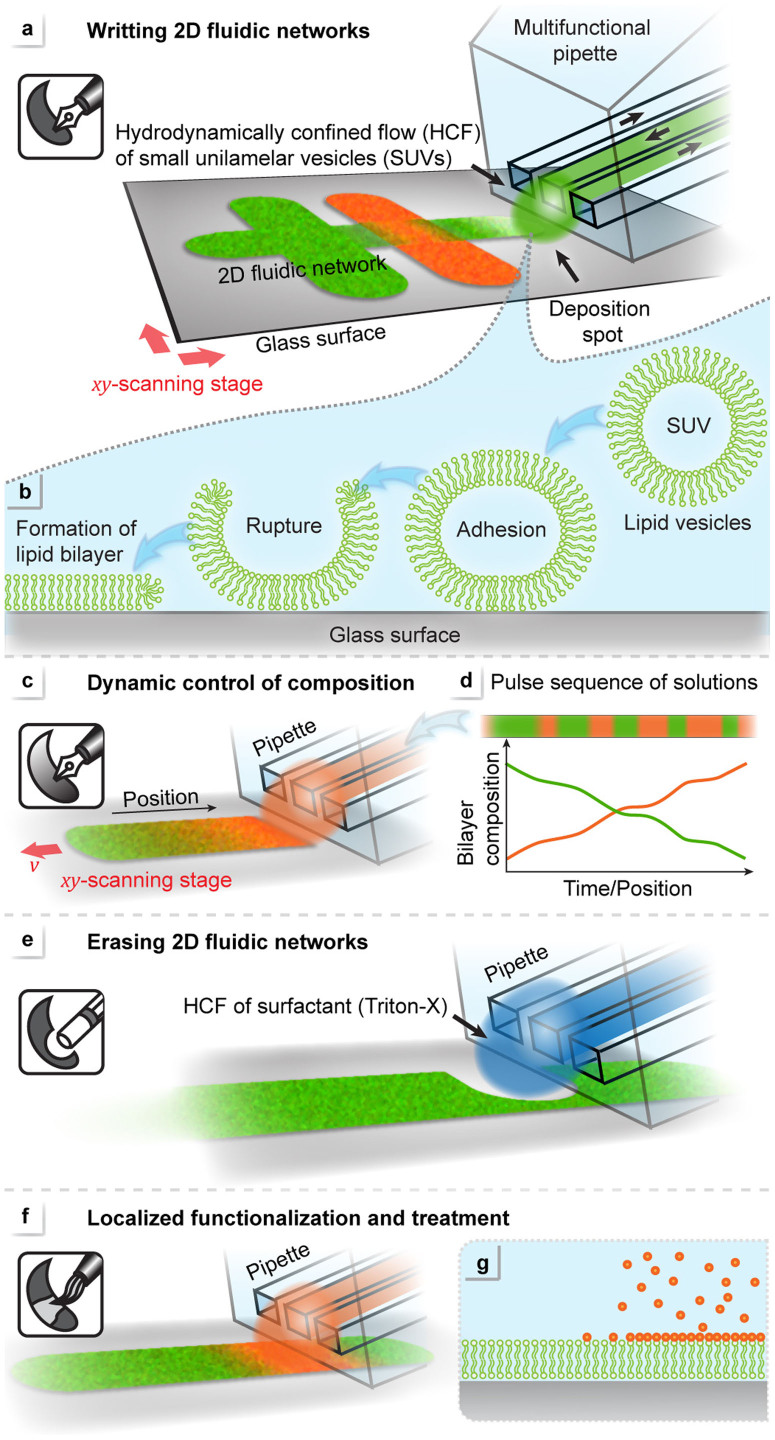
Processing tool box for the “Lab on a biomembrane”, using a virtual flow chamber at the tip of the multifunctional pipette. Up to four process steps can be consecutively applied with the device. (a) Writing 2D fluidic networks of a phospholipid bilayer by dispensing small unilamellar vesicles to a confined region on a glass surface. (b) Sequential vesicle adhesion, rupture and fusion lead to a continuous bilayer patch. (c–d) Dynamic control of film composition. The composition can be changed during writing, using a pulse code. (e) Site-selective removal (erasing) of membrane from previously written patches by means of a detergent. (f–g) Localized post-treatment of previously written membrane patches with reactive compounds.

Using a hydrodynamically confined flow device (a multifunctional pipette^25^) for dispensing suspensions of small unilamellar vesicles (SUVs, 25–50 nm in diameter) in close proximity to a planar surface, we assemble a molecular film locally by means of vesicle adhesion, and subsequent fusion. Hydrodynamic flow confinement limits the exposed area on the surface to 50–100 μm in diameter, and rapid switching between different vesicle types and auxiliary solutions allows dynamic spatiotemporal control over film composition. Figure 1 presents the four main components of the toolbox: *Writing* (Fig. 1a–b), *Dynamic control of composition* (Fig. 1c–d), *Erasing* (Fig. 1e), and *Localized lipid film modification and decoration* (Fig. 1f–g). 2D-networks are directly written by providing liposomes through the pipette, while simultaneously translating the substrate by means of a motorized stage. The maximal writing speed is generally restricted by the kinetics of film formation. Diffusively continuous fluidic networks can be produced in this way, where the topology is defined by the x, y scanning sequence, and the composition depends on the lipids supplied by the pipette. The device allows multiplexing between several different lipid (e.g. SUV) types. The formation mechanism of supported lipid bilayers from small vesicles, which is schematically depicted in Fig. 1b, has been elucidated previously^26^,^27^. Vesicles adhere to the surface, rupture, and eventually transform into a continuous bilayer. Vesicle rupture occurs either immediately upon contact with the substrate or, alternatively, after a critical concentration of surface adhered vesicles is reached. The bilayer composition can be dynamically altered during the writing process (Fig. 1c, d), owing to the on-chip multiplexing capability. A writing protocol defines lipid type and order of administration, as well as writing time and stage position. Brief exposure bursts sequentially co-deposit vesicles prepared from different lipids in a pulse width modulation (PWM) like manner^28^ (Fig. 1d), where the final composition is determined by sequence and length of the individual bursts. Since the networks are diffusively connected, the compositional diversity will be lost over time. In order to preserve the composition of a particular membrane lane, it can be temporarily or permanently cut off by means of an “eraser” tool (Fig. 1e). Hydrodynamically confined flow of a detergent solution (e.g. Triton X) from the pipette is used to locally dissolve and thus remove a part of the previously written lipid film. The eraser restores the surface, which can be overwritten with a new lipid layer at a later time. In addition to creating and removing lipid material, lanes and patches can be modified by exposure to various reagents (Fig. 1f–g).

### Writing tool

**Figure 2:**
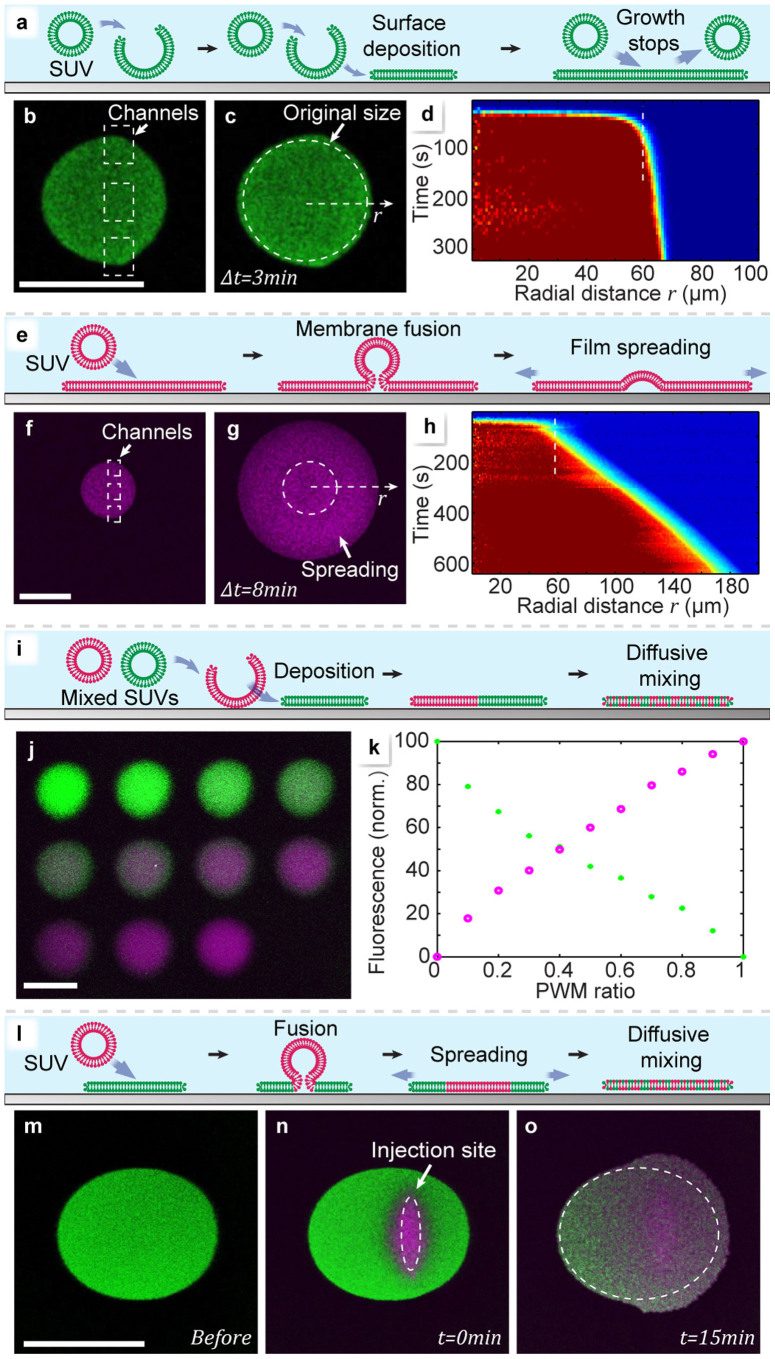
Deposition. Scale bars: 100 μm. (a) Schematic view of the vesicle adhesion-rupture mechanism (*Non-Spreading Regime*). (b) Fluorescence micrograph of a deposited POPC-488 membrane patch immediately after deposition started, and (c) after three minutes. The channel positions are marked by white rectangles in (b), and the initial patch perimeter (t = 0 min) by a white circle. in (c). (d) Fluorescence intensity depending on time and radial distance. The intensity is color coded from red (highest) to blue (lowest). (e) Schematic view of the vesicle adhesion-fusion mechanism (*Spreading Regime*). (f) Confocal fluorescence micrograph of a deposited DOTAP patch immediately after deposition started and (g) after 8 minutes. (h) Fluorescence intensity depending on time and radial distance. (i) Deposition of mixed lipid films by dispensing two vesicle suspensions sequentially by PWM-like switching (schematic view). (j) Fluorescence micrograph of eleven sequentially deposited membrane patches of gradually (10% per step) changing composition (POPC-488 (green)/POPC-655 (purple)). (k) Fluorescence intensities of the 2 fluorescent labels present in the patches. Each pair of data points (green/red) represents one individual patch in (j). (l) Fusion of SUVs with a previously deposited membrane patch, causing diffusional lipid mixing and spreading. (m) Fluorescence micrographs of a POPC-488 patch immediately after deposition, and (n) of the same patch after deposition of DOTAP-SUVs onto the center of the patch. The white line marks the injection site. (o) Fluorescence micrograph of the binary patch at t = 15 min after deposition. The white line marks the initial size of the POPC-488 patch.

**Figure 3:**
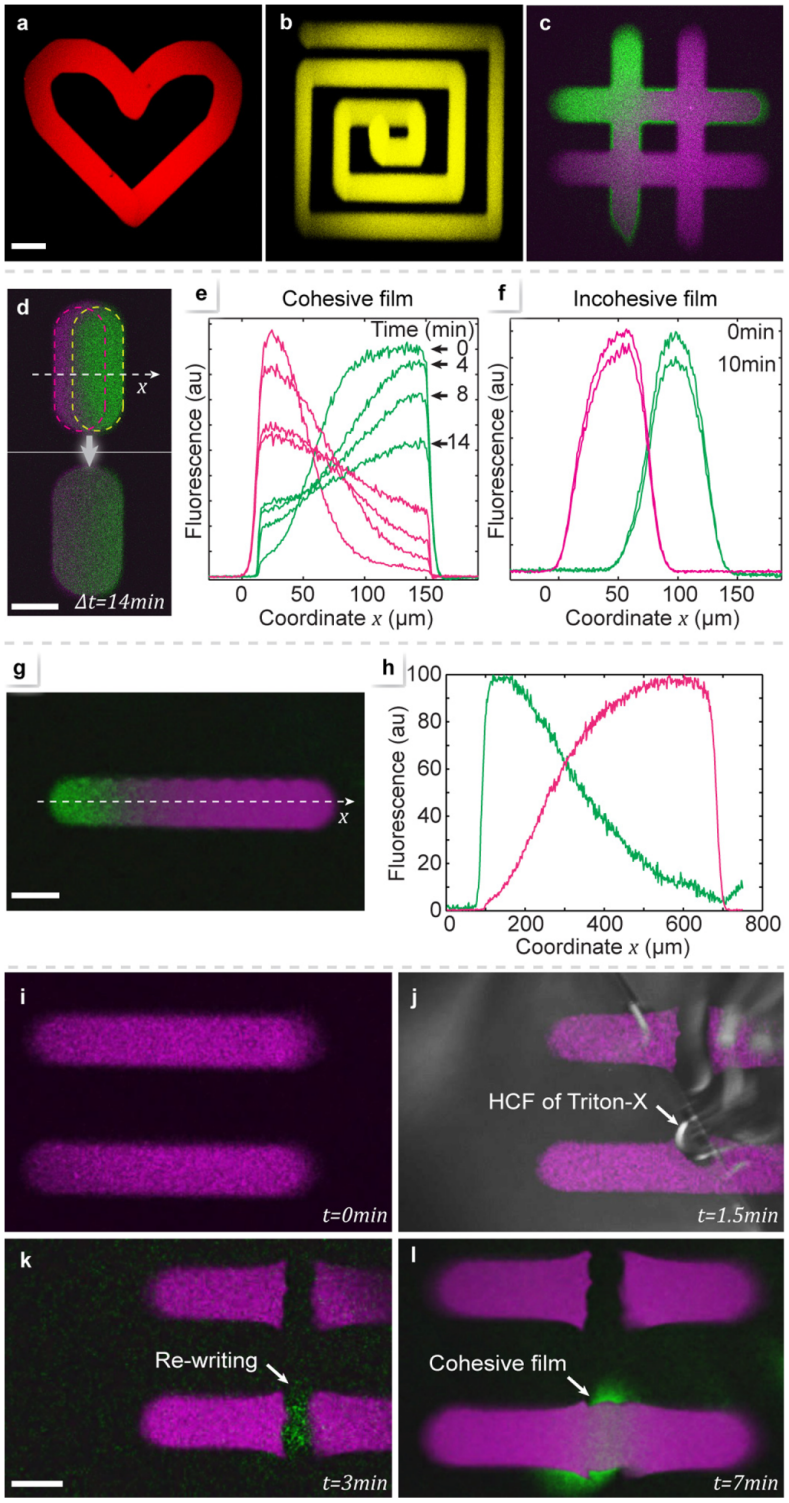
Writing, erasing, and re-writing. Scale bars: 100 μm. Confocal micrographs of a loop (a) and a connected linear array (b), which were written using POPC-655 SUVs. (c) A binary network which was written sequentially, utilizing POPC488 (green) and POPC-655 (purple), respectively. (d) Confocal micrographs of a pair of lanes, sequentially written with an offset, and composed of POPC488 (green) and POPC-655 (purple), respectively. The upper image was taken immediately after writing, the lower image after 14 min. The red and white lines in the upper picture mark the outlines of the individual lanes. (e) Fluorescence intensity profile along a coordinate across the two lanes in (d), presented for four consecutive time points after deposition. The deposited membranes are cohesive, i.e., lipids can freely diffuse between both lanes. (f) The same intensity profile for an incohesive film, where the SUV deposition time is so short that the deposited vesicles are not sufficiently dense to form a continuous bilayer membrane. (g) Confocal micrograph of lane featuring a binary composition gradient, obtained by flow switching between POPC488 (green) and POPC-655 (purple) during the writing. (h) Fluorescence intensity graph along the lane. The colors are in accordance with the image in (g). (i–l) Lane cutting and re-writing using the erasing and writing tools: (i) Confocal micrograph of two parallel lanes written with POPC-655 (purple) SUVs. (j) Overlay of the confocal transmission and fluorescence images, showing the lane cutting operation by means of the detergent Triton-X. The hydrodynamically confined flow (HCF) is clearly visible due to the higher refractive index of the triton solution. (k) Confocal micrograph depicting re-writing of the erased lower lane section with a POPC488 (green) bridge, closing the gap. (l) POPC-655 diffusion into the bridge confirms that a cohesive film is restored.

Figure 2a–h depicts the lipid film deposition. Depending on the lipid used, we observed two different regimes of adding lipid material to the forming film: non-spreading and spreading deposition. Non-spreading deposition occurs when lipid vesicles adhere to the exposed surface, rupture and fuse into a continuous lipid film^29^ (Fig. 2a). Deposition ceases upon full coverage, and the formed membrane does not grow beyond the exposed surface region. This means that overwriting of patches in the non-spreading regime is not possible. We demonstrate non-spreading deposition by using SUVs composed of 1-Palmitoyl-2-oleoylphosphatidylcholine (POPC) in Fig. 2b–c, and supplementary figure S4. The size of the generated membrane patch is largely time independent (Fig. 2d). In contrast, spreading deposition involves fusion of vesicles to an existing, previously assembled film^27^ (Fig. 2e), and the deposited membrane expands by surface spreading as long as new vesicles are supplied. Spreading deposition is achieved by using SUVs composed of L-α-phosphatidylcholine (Soy) and the cationic transfection lipid 1,2-dioleoyl-3-trimethylammonium-propane (DOTAP) (1:1 mixture, Fig. 2 f–g). Here, the radius of the membrane patch increases continuously over time for at least 600 seconds (Fig. 2h). In Fig. 2i–k we demonstrate how to change the composition of the patches dynamically during deposition (Fig. 2i). By means of the solution switching function of the pipette, two POPC vesicle suspensions, each carrying a different fluorescent label, were consecutively dispensed. In Fig. 2j, a series of 11 patches was deposited from the binary source, with the PWM ratio, i.e., composition, changing in steps of 10%. In Fig. 2k the observed fluorescence intensity of the two labels is displayed for each patch. The spreading regime of deposition can be utilized to insert membrane material into an existing patch (Fig. 2l–o). Vesicle fusion merges the supplied SUVs with a previously written membrane, first displacing the original membrane, followed by spreading and diffusional mixing (Fig. 2l). We wrote a POPC membrane patch, and subsequently exposed the center of the patch to DOTAP vesicles. Initially, the DOTAP vesicles displaced the POPC entirely from the surface, as is evident from the complete extinction of their fluorescence emission, and a slight increase of the intensity in the remaining patch. After 15 min, diffusive mixing and spreading have transformed the patch, and spreading has caused the area to increase by ~15% (Fig. 2m–o). In order to write extended two-dimensional lipid membrane structures, the deposition is combined with translation of the substrate to generate lanes. Lane writing protocols (denoted P1, P2…) for each experiment are listed in the Supplementary section S6. In Fig. 3 we demonstrate the application of the writing tool to create loops (Fig. 3a), linear arrays (Fig. 3b) and networks (Fig. 3c), consisting of one or two different lipid components. The network displayed in Fig. 3c consists of POPC with two different fluorescent labels (POPC-488, POPC-655), written sequentially from two lipid sources. It is cohesive, i.e., diffusively fully connected. Fig. 3d–e demonstrates this connectivity on a pair of overlapping lanes, which diffuse into each other within ~14 minutes. The progression of the diffusion is quantified in Fig. 3e. For comparison, Fig. 3f shows the same analysis for an incohesive film. The writing operation in combination with solution exchange was then used to create a compositional gradient along a lane of ~600 μm length (Fig. 3g). The position dependent fluorescence emission depicted in Fig. 3h gives a good estimation of the film composition, but is distorted due to apparent energy transfer between the two fluorescent labels.

### Eraser tool

The writing of lipid films is irreversible, since the transition from membrane to vesicles is energetically unfavorable, and lipid loss from a bilayer film is negligible. However, by applying a detergent to the membrane locally, it can be dissolved and permanently removed from the exposed region. Fig. 3i shows two labeled POPC lanes, which are cut perpendicularly by Triton-X exposure. In Fig. 3j the erasing has proceeded through the upper lane. The cutting edges appear sharp, only the corners of the remaining membrane are slightly deformed. After erasing, the surface appears clean and is available for re-writing. Fig. 3k is a snapshot obtained during deposition of POPC with an alternate label (green color) directly into the gap in the lower lane. After completed re-writing, the gap is repaired by a cohesive lipid film, indicated by the diffusion of the original label (red color) into the gap region (Fig. 3l).

### Functionalization tool

**Figure 4:**
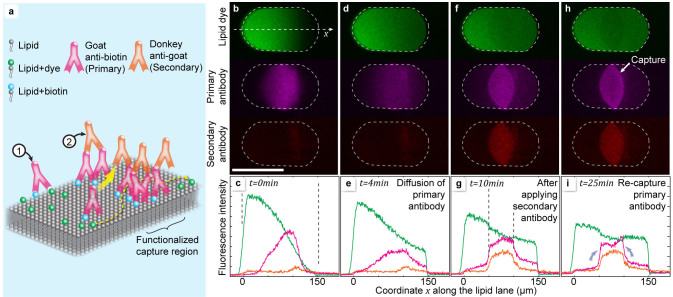
Surface functionalization. Scale bars: 100 μm. (a–i) Creation of a diffusional filter for lipid attached molecules. (a) Artist's view of the molecular arrangements the two different antibodies, each binding to two antigens, effectively cross-linking the biotinylated lipid molecules in the film. (b–e) Confocal fluorescence micrographs of the writing sequence (false-colored). Initially, a binary lipid membrane lane is sequentially written, the first half using biotin/fluorescent dye labeled POPC-488B, the second half using the non-fluorescent POPC-B. A lane of a fluorescently labeled primary antibody (goat anti-biotin) is then written perpendicularly over the center of the composite lane. Subsequently, this antibody lane is overwritten by a secondary, differently labeled anti-goat antibody, locking the primary antibodies in place. White arrows depict the writing sequence. The three fluorescent labels are simultaneously imaged in different emission channels. (f–i) Lateral fluorescence intensity profiles for the three labeled species. Green: labeled lipids, red: primary, orange: secondary antibody. Grey arrows in (i) point to an increase in the fluorescence intensity of the anti-biotin fluorophore at the borders of the central region, indicating that the primary antibody diffuses into the barrier and is being trapped there.

We here demonstrate how the writing methodology is applied to already deposited membrane lanes and networks, and describe a series of experiments involving biotin antibody recognition^30^ as an example for multistep functionalization (Fig. 4a). POPC vesicles were modified to contain 1% biotin-lipid conjugate, and utilized to write a 100 μm composite membrane lane, one half of its area fluorescently labeled. The central part of the lane was then decorated with IgG goat anti biotin antibodies (primary). After 4 minutes, which allows the antibody patch to diffusively broaden, donkey anti goat antibodies (secondary) were written over exactly the same area. We observed that the diffusion of both primary and secondary antibody is arrested. This development is shown individually for each channel in Fig. 4b–i, and quantified in the associated charts. The analysis shows that the functionalized region does not hinder diffusion of the fluorescent label, but collects, or “captures” anti biotin antibodies that have previously been diffusing out (grey arrows in Fig. 4i, Supplementary figure S15). When the second antibody is instead applied immediately after the first, avoiding diffusion, the first antibody is efficiently retained by the functionalized region (Supplementary figures S14 and S15).

## Discussion

**Figure 5:**
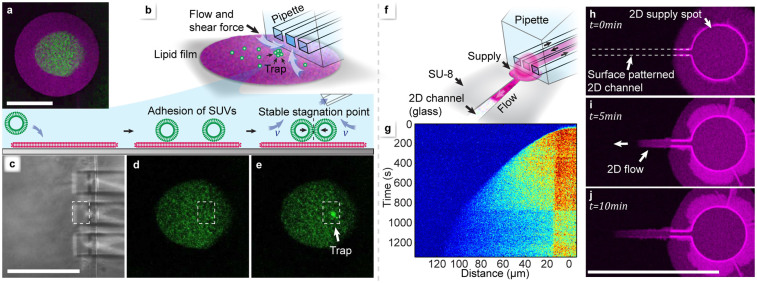
Lab on a membrane - application examples for the toolbox. Scale bars: 100 μm. (a–e) Hydrodynamic trapping of surface-associated SUVs. a) Sequential deposition of DOTAP, followed by POPC-488 vesicles, which adhere, but do not fuse with the film. (b) Switching the middle channel of the multifunctional pipette from injection to aspiration, the vesicles are drawn to the channel opening by the hydrodynamic forces (schematic view). The free in-plane, but restricted perpendicular mobility concentrates the vesicles in a stable stagnation point at the pipette tip. White arrows depict the liquid inflow. (c–e) Confocal micrographs of the trapping region. (c) Transmission image showing the pipette tip, and fluorescence images showing (d) the deposited vesicles, and (e) collection of vesicles in the stagnation point. The position of the middle channel is visualized in each image by a white rectangle. (f–j) Directed lipid spreading in 2D channels on a patterned surface. (f) Schematic view of deposition and the spreading process on a SU-8 photoresist surface, featuring a microfabricated lane which promotes lipid adhesion. The deposition device is dispensing vesicles to a circular deposition region, from where the film flows into the channel. (g) Fluorescence intensity profile depending on distance and time. The intensity is color coded from red (highest) to blue (lowest). (h–j) Confocal fluorescence micrographs showing in-lane spreading at three consecutive points in time.

The “lab on a biomembrane” concept is robust and practical, and facilitates the deployment and manipulation of supported biomembranes. The design of the networks is ultimately software-defined with respect to assembling membrane constituents and reactants, and to substrate positioning and timing. Diverse physical and chemical operations are possible, for example:

If the deposition sequence of the experiment shown in Fig. 2l, in which a POPC patch was overwritten by DOTAP vesicles, is reversed, POPC vesicles do not fuse with the DOTAP patch, but adhere to its surface and maintain some diffusional mobility (Fig. 5a). By reversing the injection flow from the middle channel of the pipette to a high aspiration rate, vesicles are collected in a stagnation point on the membrane surface beneath the channel exit (Fig. 5b–e). This previously reported hydrodynamic trapping of membrane components^31^ is possible because the particles experience the forces exerted by the aspiration flow and move laterally, but are restricted in perpendicular direction. A brief analysis of the trapping parameters is included in the supplementary section S12. Another prospective application area is directed transport of lipid film and associated molecules^16^,^17^, using patterned surfaces. We have created spreading lanes by covering the substrate with a hydrophobic polymer, where we defined accessible substrate areas photolithographically. Fig. 5f shows schematically how a circular deposition area, which is connected to a spreading lane, is accessed by the pipette. Fig. 5h–j are fluorescence recordings from a 10 minute deposition experiment with fluorescently labeled DOTAP vesicles on a surface pre-coated with POPC, demonstrating that the lipid front propagates only in the spreading lane. The sharp boundary in the distance plot of the emission intensity displayed in Fig. 5g indicates that the transport of lipid on the surface occurs through spreading, rather than by diffusion into the POPC film. This is analogous to capillary driven convective flow in 3D microfluidics^32^.

The “lab on a biomembrane” provides a multitude of experimental options at unprecedented convenience and flexibility, and has the potential to widen the application scope of molecular lipid films in the life sciences and in bioinspired engineering.

## Methods

### Vesicle preparation

A two-step procedure was followed for vesicle preparation: (I) Preparation of stock vesicle suspensions, (II) Preparation of small unilamellar vesicles. De-ionized water from a Milli-Q system (Millipore) was used for all preparations.

#### (I) Stock vesicle suspensions

For each recipe, a designated amount (see table) of lipids and lipid conjugates in chloroform were mixed and diluted with chloroform to a total concentration of 10 mg/ml. 300 μl of this solution was placed in a 10 ml round bottom flask, and the chloroform was removed in a rotary evaporator at reduced pressure (−80 kPa) over a period of 6 hours. The dry lipid film at the walls of the flask was rehydrated with 3 ml of PBS buffer containing 5 mM Trisma Base (Sigma Aldrich), 30 mM K_3_PO_4_ (Sigma Aldrich), 30 mM KH_2_PO_4_ (Sigma Aldrich), 3 mM MgSO_4_*7H_2_O (Merck) and 0.5 mM Na_2_EDTA (Sigma Aldrich). The pH was adjusted to 7.4 with H_3_PO_4_ (Sigma Aldrich). The rehydrated lipid cake was placed in the fridge (4°C) overnight. In the final step the lipid cake was sonicated at 120 W/35 kHz (Bandelin Sonorex, Germany) at room temperature for 15–30 s, to induce the formation of giant vesicles of varying, mainly multiple lamellarity.

#### (II) Small unilamellar vesicles

Small unilamellar vesicles were prepared on the day the experiments were conducted. 100 μl of the desired vesicle stock solutions were diluted (1:10) with TRIS buffer [125 mM NaCl (Sigma Aldrich), 10 mM TRIS (VWR), 1 mM Na_2_EDTA (Sigma Aldrich), adjusted to pH = 7.4 and sonicated using a Sonics & Materials Vibra Cell™ High Intensity Ultrasonic Liquid Processor (Model 501, CIAB, Chemical Instruments AB, Sweden) at 15°C for 10 minutes. The sonicated samples were subsequently ultra-centrifuged at 40,000 rpm at 15°C for 30 minutes to separate multilamellar aggregates and tip debris (Beckman TL-100 Ultracentrifuge, USA). The small unilamellar vesicles in the supernatant were transferred to a separate tube. **Critical:** For high quality membrane fabrication, the vesicle suspensions must be freshly prepared. Upon prolonged storage, vesicle preparations form agglomerates.

### The multifunctional pipette

The original multifunctional pipette with three on-chip solutions has been reported elsewhere^25^. Details on design and function of the re-engineered device used in the present study are provided as supplementary information. The pipette was operated by means of an in-house build pneumatic control unit, which interfaces to a PC computer via USB 2.0. It supports valve-less switching between four solutions, allows controlling the flow rates and adjusting the size of the hydrodynamically confined flow (HCF) volume, *i.e.*, the extent of the region to which solutions are delivered.

### Other experimental details

The preparation of plain and patterned glass surfaces is described in supplementary section S1, a detailed overview of the lipid mixtures used in experiments in section S2. Sections S3 and S4 describe the experimental setup and the microscopy methods used. Section S5 gives an overview of the control algorithm, and section S6 lists the protocols for the individual experiments. Supplementary sections S7–S14 contain additional data and experimental details for: the deposition of lipid films from single (S7) and multiple components (S8), writing (S9), diffusion measurements (S10), membrane functionalization (S11), and hydrodynamic trapping (S12), and a set of fluorescence recovery after photo-bleaching (FRAP) experiments included as alternative method for determination of the lipid diffusion constants for different deposition times. A table of literature values is also included (S13). Further, the deposition spot size dependence on the flow rate and distance of the pipette from the substrate (S14). Four supplementary videos, corresponding to figures 2–5, are also supplied. Descriptions of the videos are located at the end of the supporting section.

## Author Contributions

A.A. proposed the concept. A.A., I.G. and A.J. designed the experiments. A.A., I.G. and B.H. performed the experiments. A.A. analyzed data and performed simulations. A.A. prepared figures. A.J., A.A. and I.G. wrote the manuscript.

## Supplementary Material

Supplementary Information Supplementary Information

Supplementary Information Video 1. Deposition.

Supplementary Information Video 2. Writing and erasing.

Supplementary Information Video 3. Functionalization.

Supplementary Information Video 4. Other examples.
