# Supplementary material for: Lab on a Biomembrane: Rapid prototyping and manipulation of 2D fluidic lipid bilayers circuits
Source: Sci Rep. 2013 Sep 25;3:2743. doi: 10.1038/srep02743 (PMC3783038; doi:10.1038/srep02743)
Supplement: Supplementary Information [file srep02743-s1.pdf]

Lab on a Biomembrane

Rapid prototyping and manipulation of 2D fluidic lipid bilayers circuits

Alar Ainla, Irep Gözen, Bodil Hakonen and Aldo Jesorka

Supplementary Information

Table of contents

Table of contents 1
Supplementary Methods 2
S1. Preparation of surfaces 2
S2. Preparation of lipids and solutions 2
S3. Multifunctional pipette 3
S4. Experimental setup 3
S5. Control algorithm 4
S6. Protocols 5
Supplementary Data 6
S7. Basic deposition process 7
S8. Deposition of multiple components 7
S9. Writing 10
S10. Diffusion measurement and reference 11
S11. Functionalization 12
S12. Trapping 13
S13. Auxiliary diffusion analysis of the lipid films using FRAP 13
S14. Membrane deposition dependence on operation parameters of the pipette 16
Supplementary Videos 17

Supplementary Methods

S1. Preparation of surfaces

Glass surfaces. Circular microscope cover glasses #1.5 (Menzel-Gläser, 47mm diameter) were obtained from Thermo Scientific (Sweden). Before use, the glass surfaces were cleaned in the MC2 process laboratory at Chalmers University of Technology (class 100 semiconductor clean room), using the processing facilities, chemicals and materials provided there. First, the slides were immersed in freshly prepared piranha solution (3:1 v/v mixture of concentrated H_2SO_4 and 30% H_2O_2, heated to 100-110 °C) for 10 min, followed by rinsing with deionised water and blow drying with nitrogen. Thereafter the glass slices were mounted to a Willco Wells® dish frame using a dedicated double sided tape and assembly kit (Willco Wells B. V., Amsterdam, Netherlands) and stored in a sealed plastic bag until use. **Critical:** smooth and homogeneous surfaces are essential for the function of the 2D fluidic network. For example, sputtered silicon dioxide surfaces can be readily covered with lipids, but the forming films are not diffusively conductive and do not support transport of material.

SU-8 surfaces. The cleaned cover glasses were coated with ~2 µm high SU-8 patterns using the procedure provided by Microchem Corporation. SU-8 2002 (Microchem Corp, Massachusetts, USA) was spin-coated at 3000 rpm for 30 s, followed by soft baking for 2 min at 95 °C on a hot-plate. The SU-8 film was exposed with a dose of 120 mJ/cm^2 on a Karl-Süss contact mask aligner MA6 (G-line, 5-6 mW/cm^2), using the "Low-Vac" mode with a bright-field chromium mask. The substrates were then post-exposure-baked for 2 min at 95 °C on a hot-plate. Thereafter, the SU-8 was developed in SU-8 Developer (Microchem) for 1 min using two sequential bathes, rinsed by spraying with clean developer, and blow dried with nitrogen, yielding a SU-8 coated cover glass where the channels are formed by the exposed glass. The surfaces were plasma cleaned briefly in a Plasma Therm BatchTop RIE (50 W, 250 mTorr, 1 min) plasma chamber, and hard baked for 10 min at 200 °C on a hot plate with slow heating and cooling to prevent crack formation. The so prepared glass slides were mounted to dish frames like the plain glass slides described in the previous section.

S2. Preparation of lipids and solutions

Vesicle preparation

The compositions of the six lipid mixtures used in the experiment are summarized in the table S1.

Table S1. Lipid mixtures used in experiments (wt%).

Denomination	POPC	PC	DOTAP	ATTO 488-DOPE	ATTO 655-DOPE	Biotin-PE
**POPC-488**	99%	-	-	1%	-	-
**POPC-655**	99%	-	-	-	1%	-
**POPC-B**	99%	-	-	-	-	1%
**POPC-488B**	98%	-	-	1%	-	1%
**POPC-655B**	98%	-	-	-	1%	1%
**DOTAP-655**	-	49%	50%	-	1%	-
**DOTAP-488**	-	49%	50%	1%	-	-

Lipids: 1-Palmitoyl-2-oleoyl-*sn*-glycero-3-phosphocholine (POPC), Soy L-α-phosphatidylcholine (PC), 1,2-dioleoyl-3-trimethyl-ammonium-propane (DOTAP), 1,2-dioleoyl-*sn*-glycero-3-phosphoethanolamine-N-(cap biotinyl) (Biotin-PE) were obtained from Avanti Polar Lipids (USA). ATTO 488 1,2-dioleoyl-*sn*-glycero-3-phosphoethanolamine (ATTO 488-DOPE) and ATTO 655 1,2-dioleoyl-*sn*-glycero-3-phosphoethanolamine (ATTO 655-DOPE) were provided by ATTO-TEC GmbH (Germany). **Triton X** was obtained from Sigma Aldrich and diluted 1:10 with TRIS buffer. **Antibodies** were obtained from Agrisera (Sweden). *Goat anti-biotin antibody conjugated to DyLight 650* ("Goat anti-biotin"): 0.2 mg of goat anti-biotin antibody was dissolved in 1 ml of 10 mM TRIS buffer. *Donkey anti-Goat IgG antibody conjugated to DyLight 594* ("Anti-goat"): 0.2 mg of donkey anti-goat antibody was dissolved in 1 ml of 10 mM TRIS buffer. All antibody solutions were prepared instantly prior to the experiments, at room temperature.

S3. Multifunctional pipette

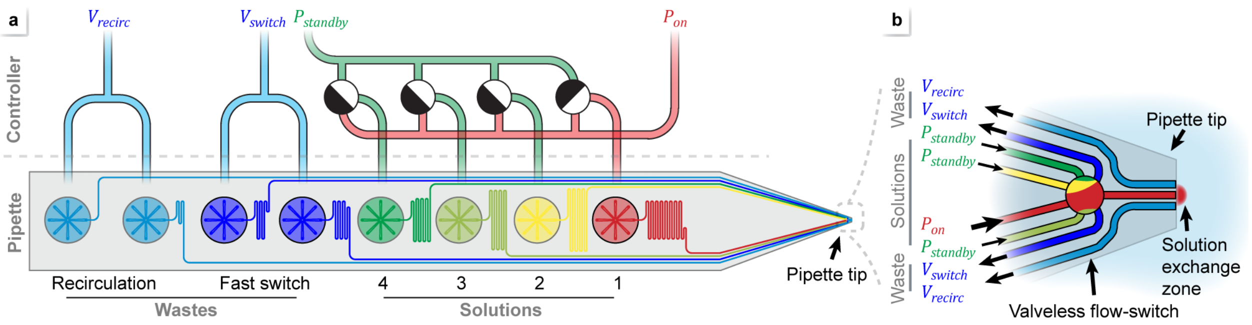

Fig S1. Layout of the multifunctional pipette. Schematics and control circuitry: (**a**) multifunctional pipette layout and external connections, and (**b**) magnification of the flow switch and pipette tip. Pressures used for operation: $V_{recirc}=V_{switch}=-100$ *mbar*, $P_{standby}=20$ *mbar*, $P_{on}=200$ *mbar*.

Table S2. Essential parameters of the four-solution pipette, and of its operation.

Parameter	Value
**Channel size**	30 µm x 30 µm
**Channel-channel separation at the tip**	20 µm
**Channel-bottom separation at the tip**	20 µm
**Solution reservoirs**	35 µL
**Flow conductance of supply channels**	53 nL/(s*bar)
**Outflow**	3.2 nL/s
**Inflow (from 2 channels)**	10.6 nL/s
**Ratio (outflow/inflow)**	0.3
**Run-time**	55-110 min
**Solution exchange time**	~100 ms

S4. Experimental setup

The experimental setup (Fig. S2) comprised the multifunctional pipette, a laser scanning confocal microscope Leica IRE2 (Leica Microsystems GmbH, Wetzlar, Germany) equipped with Leica TCS SP2 confocal scanner with AOBS™ and Ar/ArKr and HeNe lasers to provide excitation wavelength 488, 594 and 633 nm. Objectives used were HC PL APO CS 20x 0.70 UV and HCX PL APO CS 40x 1.25 OIL UV. The sample position was controlled by a scanning stage SCAN IM 120x100 (Märzhäuser Wetzlar GmbH & Co. KG, Wetzlar, Germany), equipped with a Corvus stage controller (Märzhäuser). Both scanning stage and pipette control unit were connected to a PC computer via USB port. Custom software, written in Microsoft Visual C++ (.NET), allowed simultaneous control of stage position and pipette control unit, through which the liquid composition and deposition spot size were controlled. The pipette was held and positioned in the beginning of an experiment by a 3-axis water hydraulic micromanipulator Narishige MH-5 (Japan). During the experiment, the pipette tip was positioned about ~10-20 µm above the surface, so that materials could be delivered to the surface, while avoiding direct contact, which would damage the lipid film.

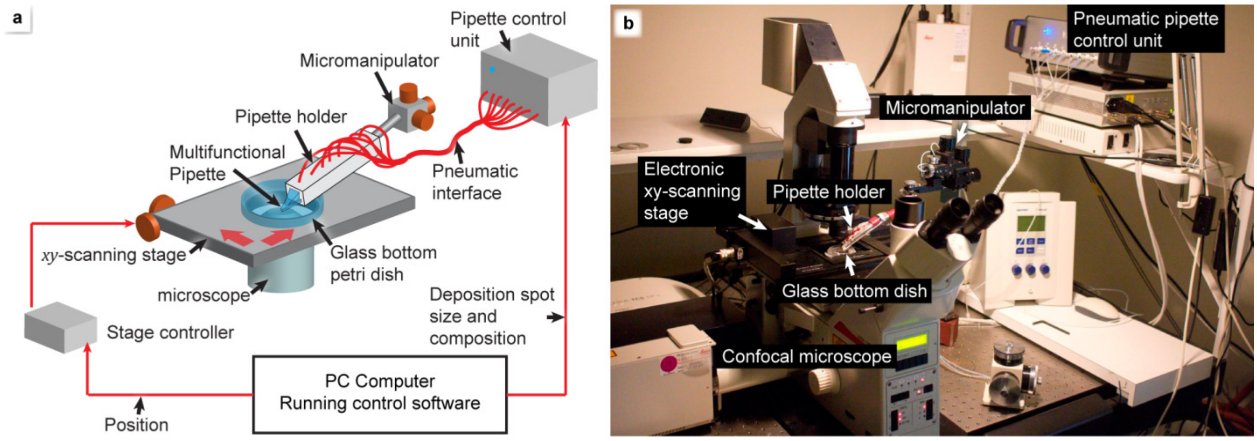

Fig S2. Experimental setup. Schematic drawing (**a**) and a photograph (**b**).

S5. Control algorithm

The algorithm used to control the writing and subsequent processing of 2D fluidic networks is illustrated in figure S3. The translation of the motorized stage, i.e., the substrate surface, occurred step-wise from one point to the next with step length L_{step}, where deposition time $t_{deposition}$ was allowed in each point to process the surface. In order to achieve a diffusively connected lane, the step length is chosen to be smaller than the spot size ($L_{step} < L_{spot}$), where L_{spot} is the diameter of the hydrodynamically confined flow (HCF) volume. If the step length is decreased, the edges of the lane become accordingly smoother. To move between unconnected parts of a network, i.e., to translate without writing, the outflow is switched off ($t_{switching}$ = 2 - 5 s). Different parts of a network can thus be created or processed with different solutions. There are up to four different solutions possible with the current system, but the number of individual solutions can be greater, depending on the pipette design.

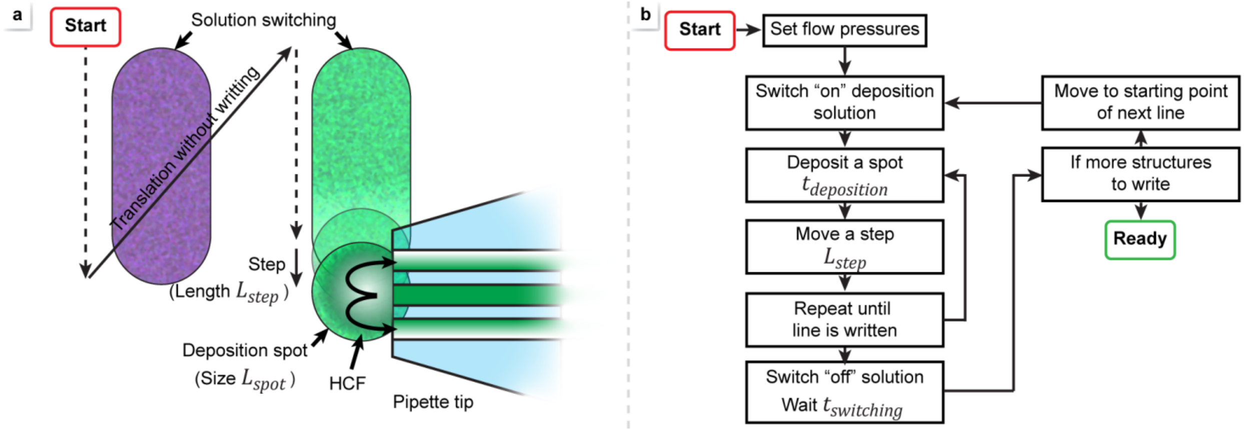

Fig S3. Control algorithm for writing and processing 2D fluidic networks. (**a**) Concept of writing two independent lanes with different lipid composition. (**b**) Block diagram of the control algorithm.

S6. Protocols

The protocols which were used in the experiments are listed in table S3.

Table S3. Experimental protocols.

Protocol	Experimental description
**P1**	**Deposition of a POPC spot.** The pipette was positioned and the flow of lipid vesicle suspension (POPC-488) was switched-on. Density and size evolution of the spot were analysed.
**P2**	**Deposition kinetics and density of the POPC spot.** The pipette was positioned and the flow of lipid vesicle suspension (POPC-655) was switched-on. Kinetics of the density change was analyzed.
**P3**	**Deposition of a DOTAP spot.** The pipette was positioned and the flow of lipid vesicle suspension (DOTAP-655) was switched on. Density and size evolution of the deposition spot were analysed.
**P4**	**On-surface dilution series of POPC with different labels.** The pipette was loaded with two different types of lipid vesicles (POPC-488 and POPC-655). Dilutions were created using the pulse width modulation (PWM) principle on the surface. For this, the deposition was multiplexed between the two sources, where the PWM period was 10 s. Each spot was deposited for 30 s, thus each spot deposition contained three periods. In total 11 spots were created with the duty cycle changing in 1 second steps (e.g. 10:0, 9:1, 8:2, 0:10 seconds). The spots with a diameter of 100 µm were created as a 3 x 4 grid with a pitch of 300 µm. The stage movement between the spots was controlled by the protocol software.
**P5**	**Fusion of DOTAP into a POPC spot.** The pipette was loaded with two types of lipid vesicles (POPC-488 and DOTAP-655). Thereafter the pipette was positioned, and the flow of POPC-488 vesicle suspension was switched on for 60s to deposit a POPC spot. Thereafter, the flow was switched off and the pressure conditions were set such that the outflow rate would be about half the previous rate, to ensure that the size of the hydrodynamically confined flow (HCF) volume is reduced and the DOTAP is deposited within the boundaries of the already existing POPC film. Thereafter DOTAP-655 deposition was switched-on.
**P6**	**Overlapping DOTAP-on-POPC spot.** This experiment was performed in a manner similar to the previous one, except that the flow-conditions were not adjusted between the different lipid spots. However, the pipette position was moved slightly by ~30 µm to create two partly overlapping films.
**P7**	**Deposition of POPC onto a DOTAP spot.** This experiment was performed similarly to **P5**, but the deposition sequence was exchanged. First DOTAP-655 was deposited for 60s, followed by POPC-488 for 90s. Since the area of the initially deposited DOTAP-655 film grows due to spreading, there was no need to reduce the size of the HCF volume between different lipid types.
**P8**	**Overlapping POPC-on-DOTAP spot.** This experiment was performed similarly to **P6**, but the deposition sequence was exchanged, such that a DOTAP-655 spot was deposited first. Subsequently, after the pipette was positioned to the edge of the spot, POPC-488 was deposited.
**P9**	**Writing a loop.** The pipette was positioned and the flow of lipid vesicles (POPC-655) was switched-on. A "heart" shaped closed loop was written using step length $L_{step} = 13 - 18\ \mu m$ and step deposition time $t_{deposition} = 7\ s$. Since the spot diameter was about 100 µm, the line area becomes deposited in about 30 s. Total writing: 200 steps, corresponding to ~24 min writing time.
**P10**	**Writing a "Labyrinth".** Similar to **P9**, with $L_{step} = 13\ \mu m$ and $t_{deposition} = 3\ s$. Total: 560 steps and a writing time of ~28 min.
**P11**	**Writing a two-component network 1 (grid).** The pipette was loaded with two types of lipid vesicles (POPC-488 and POPC-655). Four lines, two vertical and two horizontal, were sequentially written, out of which one vertical and one horizontal line were written from POPC-488, and the other two from POPC-655. $L_{step} = 50\ \mu m$ and $t_{deposition} = 10\ s$. Total writing time was ~15 min, followed by 15 min time for diffusion.
**P12**	**Writing a two-component network 2 (cross).** This experiment was performed similarly to **P11**, using a different cross geometry, composed of two lines, a vertical (POPC-488) and a horizontal (POPC-655) one.
**P13**	**Diffusion of two dyes in a POPC film 1.** The pipette was loaded with two types of lipid vesicles (POPC-488 and POPC-655) and positioned. Two 300µm long and 100 µm wide lanes were written ($L_{step} = 25\ \mu m$ and $t_{deposition} = 10\ s$). First POPC-488 was written, followed by POPC-655. The lanes were offset by 50 µm and were overlapping partially. After writing, the diffusion was monitored for 15 min.
**P14**	**Diffusion of two dyes in a POPC film 2.** This experiment was performed similarly to **P13**, but the offset distance between the lanes was increased to 120 µm. The diffusion was monitored for 8 min.
**P15**	**Writing a gradient.** This experiment was performed similarly to **P4**, but instead of depositing individual spots, the lipid vesicles were deposited gradually on a single lane in 80 µm steps, creating a smooth gradient.

**P16**	**Erasing.** The pipette was loaded with three solutions: two types of lipid vesicles (POPC-488 and POPC-655) and a solution of dilute Triton-X. Two parallel lanes of 150 µm length were written, using POPC-655. Thereafter the outflow was switched off and the supply pressure P_{on} was increased from 200 mbar to 250 mbar, since Triton-X is more viscous. Thereafter Triton-X solution was switched on. The HCF volume is easily visible in the transmission channel of the microscope, due to a higher refractive index (Triton-X has a refractive index of n = 1.49 vs. n = 1.33 for water). Triton-X was scanned perpendicularly over the lower lane. Thereafter the outflow was switched off again, P_{on} was set to its initial value, and the pipette was positioned onto the cutting point of the lane. A spot of POPC-488 was deposited in order to reconnect the lane. The first (upper) lane served as a reference.
**P17**	**Diffusion of a dye.** The pipette was loaded with two types of lipid vesicles (POPC-488B and POPC-B) and positioned. A 200 µm lane was written ($L_{step} = 10$ µm and $t_{deposition} = 5\ s$), such that during the first half of the deposition (0-100 µm) POPC-488B was deposited, and during the second half (100-200 µm) POPC-B. Thereafter the diffusion was monitored for about 15min. Note that due to the round lane ends, 200 µm scanning length results in a 300 µm lane.
**P18**	**Functionalization Sequence 1.** The pipette was loaded with two types of lipid vesicles (POPC-488B and POPC-B) and two antibody solutions: goat anti-biotin, and anti-goat. The pipette was positioned and a 200 µm lane was written ($L_{step} = 10$ µm and $t_{deposition} = 5\ s$), such that first 100 µm were composed of POPC-488B, followed by 100 µm of POPC-B. Thereafter the pipette was moved 100 µm backwards onto the middle point of the lane, and goat anti-biotin was applied for 5 s, followed by 10 min diffusion time. Thereafter anti-goat was applied for 5 s onto the same spot, after which re-distribution of molecular species was monitored for about 15 min.
**P19**	**Functionalization Sequence 2.** This experiment was performed similarly to **P18**, but the 10 min diffusion time after functionalization with goat anti-biotin was omitted, and anti-goat was applied directly after goat anti-biotin.
**P20**	**Trap.** This experiment was started as described in **P7**, by depositing POPC vesicles onto a DOTAP film. After POPC vesicles were deposited onto a DOTAP film, the supply pressures were adjusted (V_{recirc}=0 mbar, V_{switch}=-350 mbar, $P_{standby}$= P_{on}=20 mbar) for maximal inflow ($Q \approx 33\ nl/s$) through the middle channel. Trap formation and collection of vesicles under the middle channel were monitored.
**P21**	**Flow in patterned 2D channels.** The pipette was loaded with two types of lipid vesicles (POPC-488 and DOTAP-655) and positioned onto the patterned area. First, the surface of the patterned channel was covered with POPC-488, thereafter DOTAP-655 was deposited onto the circular supply area, and transport of fluorescently labelled lipid from the supply area into the channel was monitored for about 25 min.

In each experiment the response was recorded as a series of fluorescent confocal microscopy images, collected in up to three different fluorescence emission channels corresponding to the fluorophores used (Table S4), A brightfield transmission image was also recorded.

Table S4. Dyes and wavelengths used for excitation and collection of fluorescence.

Compound(s)	Excitation (nm)	Collected emission (nm)
**POPC-488, POPC-488B, DOTAP-488**	488	496-586
**Donkey anti-goat (DyLight 594)**	594	610-620
**POPC-655, POPC-655B, DOTAP-655, Goat anti-biotin (DyLight 650)**	633	650-800

Supplementary Data

All microscopy images presented in following section are false coloured. The actual collection ranges of emitted light, controlled by the confocal microscope, are given in Table S4 above.

S7. Basic deposition process

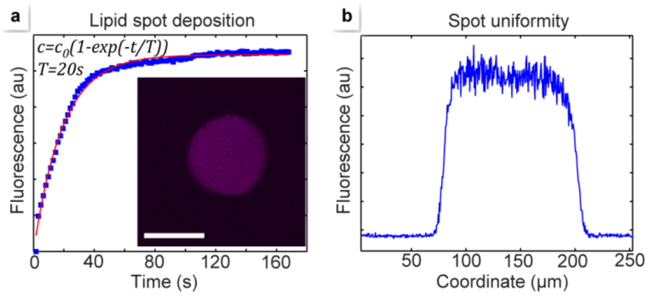

Fig S4. Spot deposition of lipid film according to protocol **P2**. (**a**) Exponential surface coverage kinetics with a time constant of about 20s. (**b**) Spot uniformity. When the surface becomes covered it forms eventually a uniform film. We observed neither deposition of multiple layers, nor further significant adhesion of lipid vesicles to the membrane.

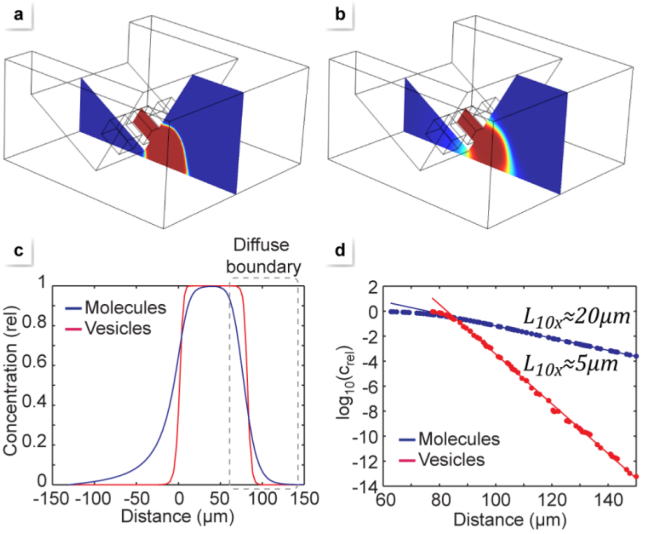

Fig S5. Simulation of diffusion for the deposition of lipid films and film functionalization. Comparison of vesicles (the size of the SUVs is about 20-50 nm in diameter) $D \approx 2 \cdot 10^{-11}\, m^2/s$ (**a**) and small molecules, like fluorescent dyes, $D \approx 5 \cdot 10^{-10} m^2/s$ (**b**), which gives a 25 fold difference in Péclet number. Vesicles are confined with a much sharper boundary than small molecules at the same flow rate and HCF volume size (**c**). In a diffuse boundary the concentration drops exponentially (**d**). 10-fold reduction in concentration occurs over characteristic length scales: $L_{10x} \approx 5\ \mu m$ and $L_{10x} \approx 20\ \mu m$, for lipid vesicles and small molecules, respectively. The flow rates used for the simulations are the same as for the experiments (Table S1). Simulations were performed with COMSOL Multiphysics 4.1, combining models of laminar flow (spf) and transport of dilute species (chds) in stationary 3D models. The viscosity of water was 1 mP*s. The pipette was positioned 10 µm above the surface at an angle of 30°. Due to a sharp chemical boundary at high *Pé*, an about 10x finer mesh was used for the diffusion calculation compared to the flow calculation.

S8. Deposition of multiple components

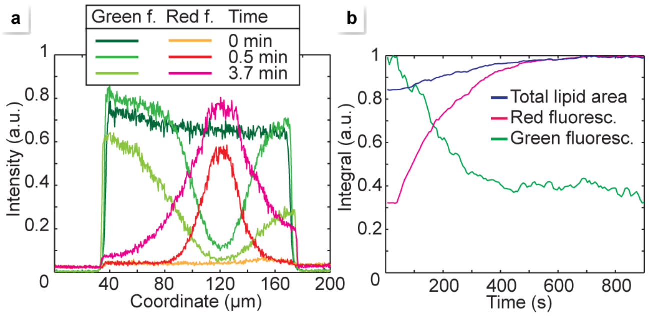

Fig S6. Depositing DOTAP on top of a POPC film (Protocol **P5**). Intensity profiles before and after injection of DOTAP (**a**). Integrals of total film area and fluorescence in two channels (**b**). Microscopy images are provided in Fig. 2 of the main text. Already a small scale injection of DOTAP-655 (area increase only about ~15%) causes significant quenching of green (POPC-488) fluorescence (about -60%). However, a local fluorescence increase of the green fluorescence can be observed outside the injection site directly after injection. This can be due to an initial compression of the film. The net decrease of the fluorescence is due to energy transfer from the green (488) to the red (655) dye. We speculate that the dyes are forming a molecular assembly, where energy from many green flurophores is efficiently funnelled to a single red flurophore, since small amounts of Atto-655 dye quench the fluorescence of many Atto-488 dye molecules. Alternatively, it could be possible that the film remains compressed and the amount of actually incorporated DOTAP is higher than predicted by the total area increase of the lipid film.

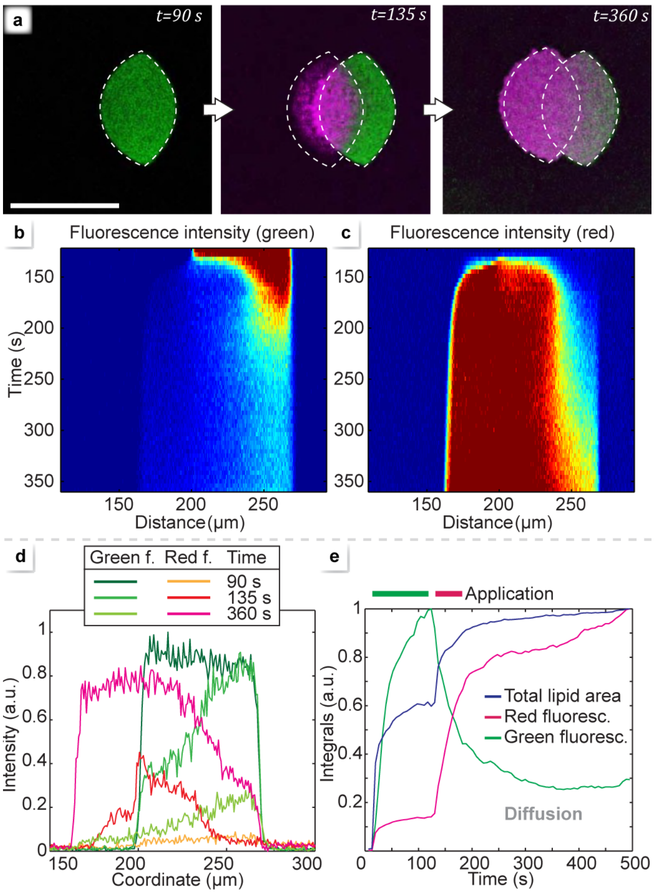

Fig S7. Overlapping DOTAP spot on a POPC spot (Protocol **P6**). Confocal microscopy images (**a**), time evolution of the fluorescence profile across the membrane patches (**b-d**), as well as the integrals of the total film area and fluorescence in both channels (**e**). DOTAP is readily depositing on the glass surface and fusing into a POPC film. Immediately after DOTAP-655 deposition has started (*t=135 s*), the red (655) fluorescence increases more rapidly on top of the POPC-488 film near its boundary, while the green (488) fluorescence is decreasing there. This supports the hypothesis of energy transfer between these dyes. The mixing is most efficient directly on the edge, where a sharp spike can be seen. Further evidence for energy transfer is provided by the integrals. After applying DOTAP, mixing between DOTAP-655 and POPC-488 occurs, which results in rapid decrease in green and increase in red florescence. Even after switching off the deposition, the membrane area increases slightly along with increasing red florescence. This can be due to on-going spreading and diffusion. Evaluating the areas, the membrane should be composed of 60% of POPC-488 (green) and 40% of DOTAP-655 (red) lipids. However, mixing with the red dye results in quenching of about 70% of the green fluorescence. The red fluorescence of ATTO-655, collected between 650 – 800 nm, is represented by purple false colour. The green fluorescence of ATTO-488, collected between 496 – 586 nm, is represented by green false colour.

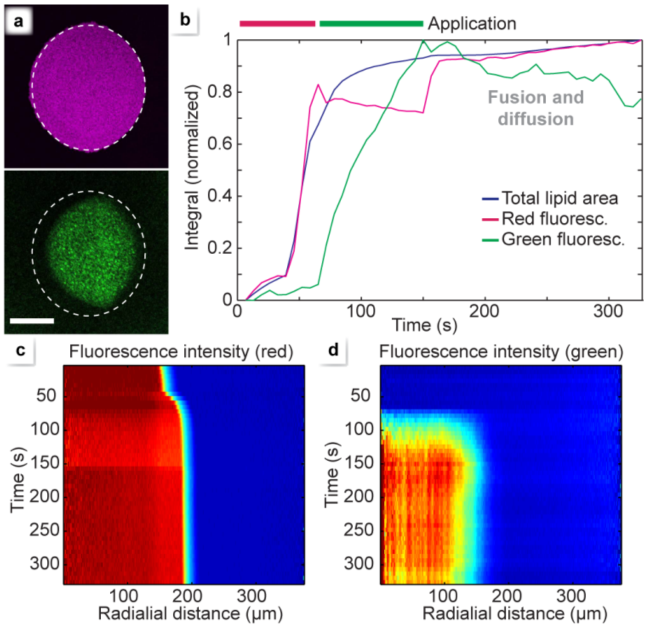

Fig S8. Deposition of POPC on the DOTAP spot (Protocol **P7**). Confocal microscopy images (**a**), integrals of total film area and fluorescence in the two channels (**b**), and time evolution of the profile of fluorescence intensities (**c-d**). These experiments indicate that POPC vesicles are adhering to the DOTAP film, but do not fuse readily, which should result in an area increase, or at least in diffusion and energy transfer. Instead, deposition of POPC results in a very small area increase (<20%), which could be also due to ongoing spreading of DOTAP. There is almost no energy transfer discernible. Note that the step in the red channel between 50 and 150 s during the application of POPC-488 is an artefact caused by mechanical interaction of the pipette and the dish, as well as slight mechanical motion caused by switching. Minor fusion is distinguishable by a slight area increase, as well as an increase in red, and decrease in green fluorescence (<10%). The red fluorescence of ATTO-655, collected between 650 – 800 nm, is represented by purple false colour. The green fluorescence of ATTO-488, collected between 496 – 586 nm, is represented by green false colour.

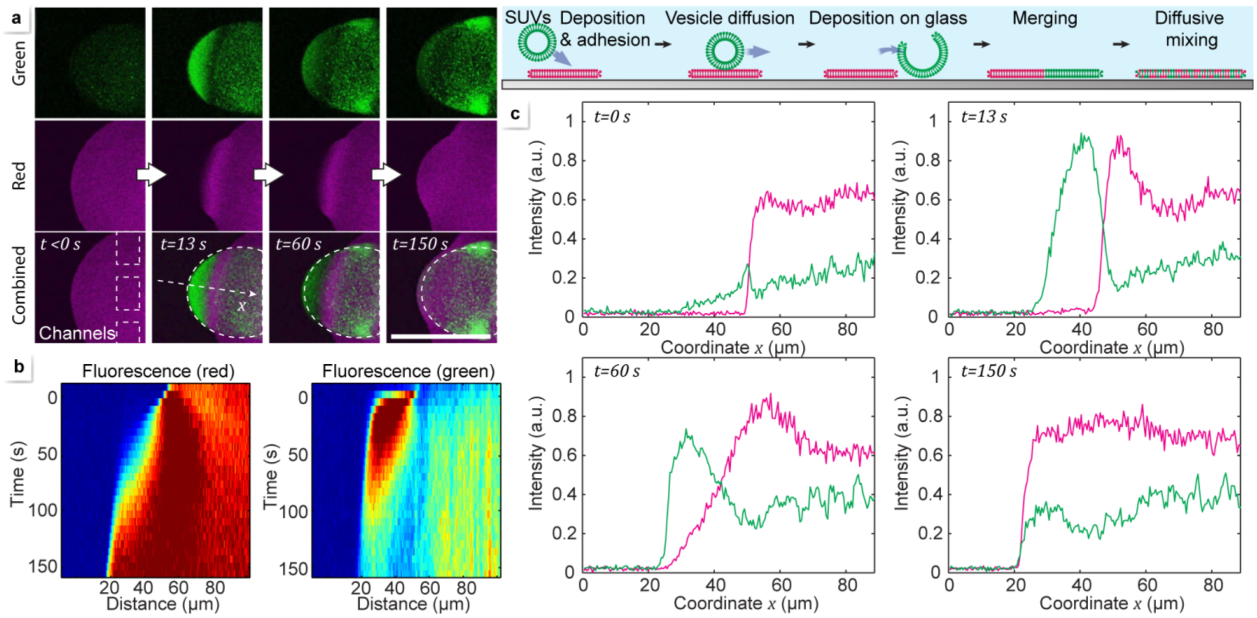

Fig S9. Overlapping POPC-on-DOTAP spot (Protocol **P8**). Confocal microscopy images (**a**), and the evolution of the fluorescence profile (**b-c**). Directly after the deposition of POPC-488 was started (*t=0 s*), a sharp increase in the green (488) fluorescence channel can be observed on the glass side of the boundary of the lipid films, as POPC vesicles immediately adhere and fuse there. This forming POPC film is diffusively connected to the DOTAP patch. This results in energy transfer and a sharp increase in red (655) fluorescence on the edge of the DOTAP patch (*t=13 s*). As diffusion progresses, the green florescence is reduced (*t=60 s*), and eventually both parts of the film are homogeneously mixed (*t=150 s*). In contrast, a darker rim is visible in the green channel on the lipid side of the boundary, indicating a loss of vesicles after deposition. POPC vesicles do not fuse with the DOTAP membrane, but maintain mobility after adhering. They are rapidly affected by the pipette inflow and removed from the patch perimeter. The accumulation of green vesicles on both sides under the inlet channels are indicators for a hydrodynamic trap, a phenomenon which was further

investigated in another experiment (see **P20, S12**). The red fluorescence of ATTO-655, collected between 650 – 800 nm, is represented by purple false colour. The green fluorescence of ATTO-488, collected between 496 – 586 nm, is represented by green false colour.

S9. Writing

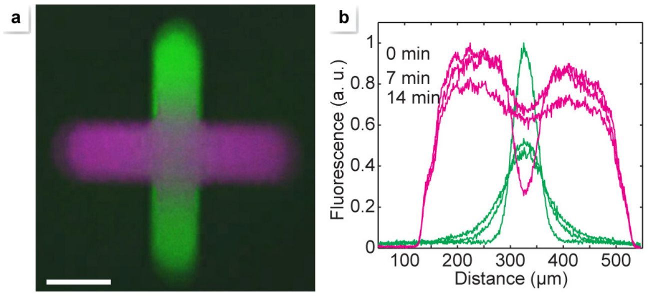

Fig S10. Writing a two-component network (Protocol **P12**). Confocal microscopy image after deposition and diffusion (**a**), and fluorescent intensity profile along the red axis of the cross (**b**). First the green (POPC-488) lane is written, which is then crossed by the red (POPC-655) lane. Since POPC films cannot be overwritten, the green lane initially causes a break in the red lane, but as they are diffusively connected, mixing occurs over time. The red fluorescence of ATTO-655, collected between 650 – 800 nm, is represented by purple false colour. The green fluorescence of ATTO-488, collected between 496 – 586 nm, is represented by green false colour.

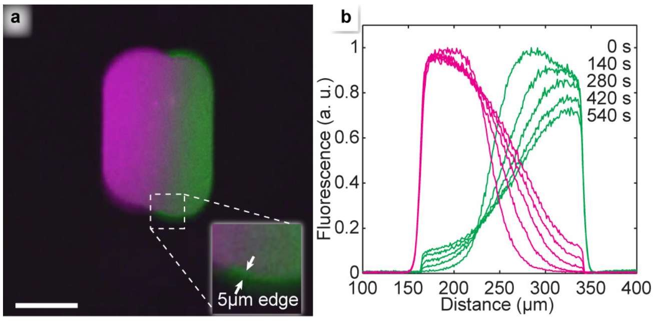

Fig S11. Diffusion of two dyes in a POPC film (Protocol **P14**). Confocal microscopy image after diffusion (**a**) and fluorescent intensity profiles across the lanes at different time points (**b**). The diffusive mixing of two lipid-anchored dyes (488 and 655) can be monitored. However, a closer look reveals that a narrow (about 5 µm) stripe at the edge of the patch is not diffusively connected (inset of panel a, and lower left and right corners in the graph in b). This is explained by the diffusive boundary of the HCF volume, where a 5 µm distance would correspond to about one order of magnitude in concentration drop (Fig. S5). While this lower vesicle concentration is sufficient to create a visible coverage with nanoscopic lipid patches, the density is too low to form a connected and cohesive film which would support diffusive transport. The red fluorescence of ATTO-655, collected between 650 – 800 nm, is represented by purple false colour. The green fluorescence of ATTO-488, collected between 496 – 586 nm, is represented by green false colour.

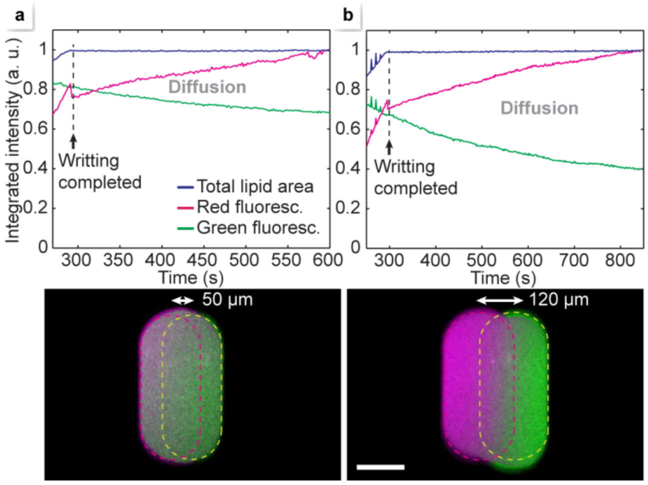

Fig S12. Integrated fluorescence intensities and normalized lipid film areas. (**a**) 50 µm offset between two lanes, in which case the film is composed of 80% of green lipid and 20% of red lipid. (**b**) 120 µm offset between two lanes, in which case the film is composed of 55% of green lipid and 45% of red lipid. During mixing there is no further deposition of lipids and the area is preserved (no spreading). However, the integrated intensities are changing such that the intensity of the green fluorescence drops while that of the red increases. This can be explained by energy transfer from the green absorbing (488 nm) to the red absorbing (655 nm) dye. The red fluorescence of ATTO-655, collected between 650 – 800 nm, is represented by purple false colour. The green fluorescence of ATTO-488, collected between 496 – 586 nm, is represented by green false colour.

S10. Diffusion measurement and reference

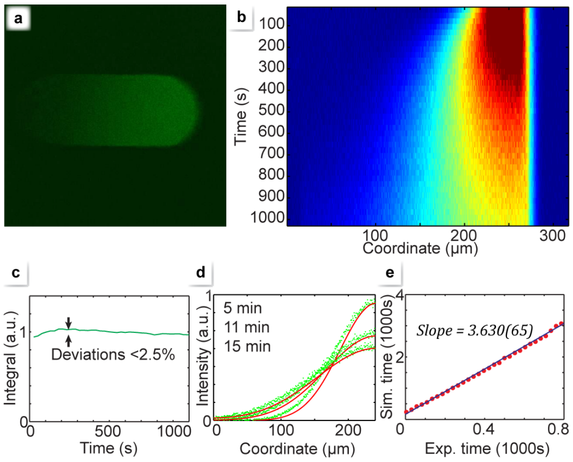

Fig S13. Diffusion of one dye (Protocol **P17**). Microscopy image (**a**). Evolution of the fluorescence intensity along the lane (**b**). Integrated fluorescence intensity over the total area of the lane (**c**). Fitting simulation (red line) and experiment (green dots) (**d**). Correlation of time required in simulation and experiment for the same dye distribution (**e**). In case of a single dye, where there is no possibility for energy transfer, the integral of fluorescence intensity is well preserved with small drifts of <2.5% (std), which are caused by a combination of the stability of the microscope focus, laser intensity, detector gain and photobleaching, which is most significant in case of the low wavelength (488 nm) dye. This stability for a single dye clearly suggests that the observed large changes in other cases are due to the interactions between fluorophores. This allows us to assume that fluorescence intensity is directly proportional to fluorophore concentration and can be used to characterize diffusion. The diffusion constant was estimated using a numerical analysis. The lane was considered as a one dimensional system and a respective model was constructed in COMSOL. The initial concentration distribution was taken from the experiment and a transient simulation was used with $D_{sim} = 1\ \mu m^2/s$. The output (time series of concentration profiles) was transferred to MATLAB. Since the solution of the diffusion equation depends on the parameter Dt, the output of the simulations can be fitted to the experimental results (d). The observed slight deviations are most likely due to the fact that the lane, having round corners, is not perfectly one dimensional. When the best fitting simulation time is plotted against the experimental time, a linear relation is formed, in which the slope contains the actual diffusion constant. Since $c(D_{sim}t_{sim}) = c(D_{exp}t_{exp})$, $D_{exp} = D_{sim}t_{sim}/t_{exp} = D_{sim} \cdot Slope$. In this case $D_{exp} = 3.630(65)\ \mu m^2/s$.

S11. Functionalization

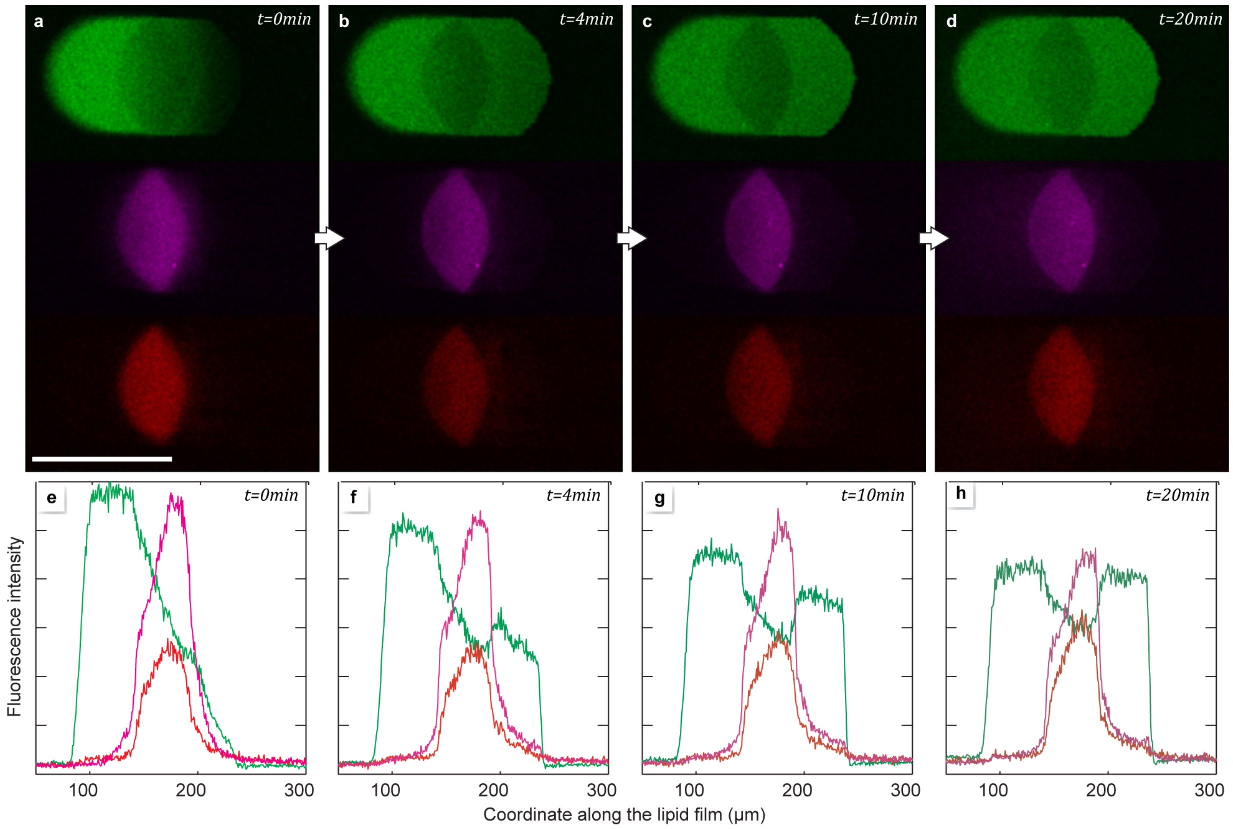

Fig S14. Functionalization (Protocol **P19**). When a secondary antibody (anti-goat) is applied directly after the primary (anti-biotin), there is essentially no diffusion of the primary antibody observable, and the distribution of both antibodies remains constant, with only minor diffusion around the edges. However, the antibody functionalized region is permeable for the lipid dye (488), which diffuses freely through the barrier. Reduced intensity of the fluorescence of POPC-488B is due to energy transfer to the antibody-conjugated dye. The graph colors correspond to the colors in the confocal micrograph. Green: POPC-488B; purple: goat anti-biotin; red: anti-goat.

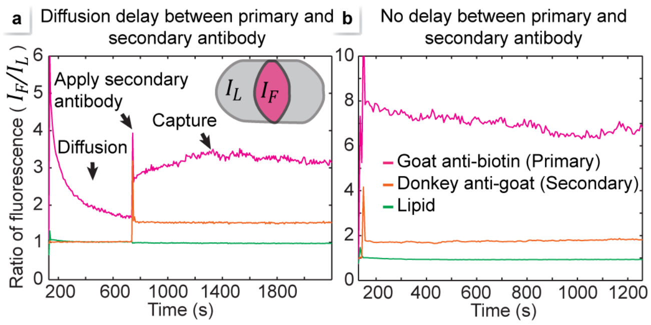

Fig S15. Ratios of average fluorescent intensities (I_F/I_L) measured on the entire lipid lane, where I_F is the fluorescence intensity measured on the functionalized region, and I_L on the remaining lane area. This ratio is a good indicator of diffusion and capture of material in the functionalized region. (**a**) Initially, a 600 s diffusion time was used in between the deposition steps of the two antibodies. During this time the primary antibody diffused out of the functionalized region, and intensity differences were equilibrating (from I_F/I_L = 4.5 to 1.5). The fluorescent intensity corresponding to the primary antibody started to gradually increase after functionalization with the secondary antibody, indicating that the anti-biotin is captured in the functionalized region. An abrupt change of the fluorescent intensity is due to cross-talk between channels. If the channel cross-talk is subtracted, the fluorescence intensity ratio was found to increase ~41%. The average intensity ratio of the secondary antibody remains constant, indicating no diffusion. Even though diffusing, the intensity ratio of the lipid remains constant due to the symmetrical deposition of the lane. (**b**) In a second experiment, the diffusion time between primary and secondary antibody was omitted. In this case the primary antibody was bound immediately, and no significant diffusion was observed. During this time, the intensity ratio decreased only by 16%.

S12. Trapping

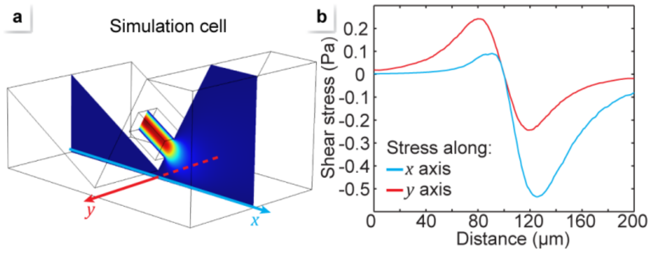

Fig S16. Hydrodynamic trap simulation to estimate the trapping force. The simulation cell (**a**) and the hydrodynamically generated stress profile on the surface (**b**). In this simulation, the pipette was positioned 10 μm above the surface at a 30° angle. A flow rate of ~ 33 nl/s was applied. For 20 to 50 nm vesicles, the estimated trapping force is in the range of 0.5 to 5 fN, at a maximum shear stress of ~0.5 Pa. The trapping force scales ∝ Q and ∝ d^2, where Q is the flow rate and d is the vesicle/particle size.

S13. Auxiliary diffusion analysis of the lipid films using FRAP

In order to determine the transport properties of different membranes and their dependence on basic fabrication parameters, and deposition time, a diffusion analysis using fluorescence recovery after photobleaching (FRAP) was employed. FRAP experiments were performed on the Leica confocal microscope described in the S4, using the FRAP module in the Leica confocal software, a 40x 1.25 oil objective and 488 nm excitation light. In all experiments, ATTO-488 labelled lipids were used. The imaging frame rate was 0.51 fps over 187.5 μm x 187.5 μm area. Firstly, 10 pre-bleach frames were collected, followed by 4 bleaching frames, during which the scanning area was reduced to 20 μm x 20 μm and laser power was increased 5-folds using the AOTF, which results in about a 440x increase in light intensity during bleaching compared to imaging. Directly after bleaching the fluorescence intensity had dropped to about 10% of its initial value. The power was then reduced, the observation area increased again, and 50 frames were collected in order to follow the fluorescence intensity recovery. In order to determine the diffusion properties, the intensity development was compared with a model (Fig S17). The normalized experimental recovery was calculated using the following formula

$$Recovery_{experimental}\ (t) = \frac{I_{bleached}(t) - I_{bleached}(0)}{I_{reference}(t) - I_{bleached}(0)}$$

It is zero at $t = 0$, and becomes 1, after complete recovery. However, the lipid film can also contain none-diffusive parts. The diffusive part or *mobile fraction* is denoted with m

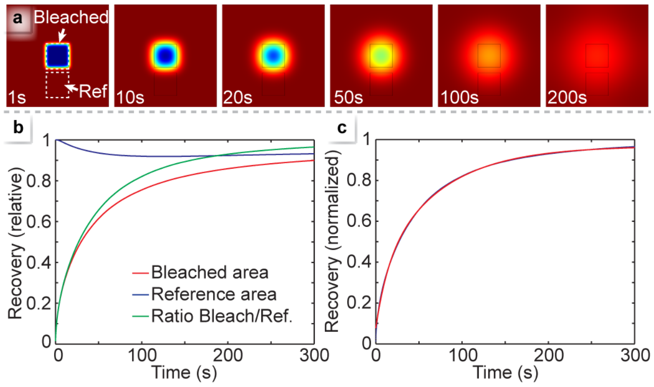

Fig S17. FRAP model. The model to fit the collected FRAP data was developed empirically from a finite element model in COMSOL, using 2D time dependent diffusion. The lipid patch was modelled as a 90 μm x 90 μm square area with closed boundaries, where the bleached area (20 μm x 20 μm) was placed in the canter. Initial concentrations inside and outside the bleached area were 0 and 1, and the diffusion constant was assumed to be $D_{model} = 1\ \mu m^2/s$. The resulting time series of the concentration profiles is shown in (**a**). In order to accommodate the reduced effect of bleaching and other sources of general intensity variations during the recovery time, the normalized intensity (intensity in the bleached area relative to the intensity in the defined reference area) was used instead of the directly measured intensity (**b**). The data from this model was fitted to a two-component exponential decay, giving an excellent fit (**c**):

$$Recovery_{model}\ (t) = a - b \cdot e^{-ct} - d \cdot e^{-et}$$

With parameters given in following:

Parameter	Value	Uncertainty (95%)	Uncertainty %
a	0.9687	4.6e-4	0.047%
b	0.6228	0.0034	0.55%
c	0.01425	8.5e-5	0.60%
d	0.2686	0.0034	1.3%
e	0.07304	0.0013	1.8%

The experimental recovery is fitted to the model:

$$Recovery_{experimental}\,(t) = m \cdot Recovery_{model}\big(k(t - t_0)\big)$$

Three parameters were determined: the mobile fraction m, the temporal scaling constant $k = D_{experimental}/D_{model}$, which allows to extract the experimental diffusion constant, and t_0, which is the time offset correction, as bleaching itself has a finite duration. Results of the FRAP experiments are shown in figure S18 for both POPC and DOTAP films, depending on different spot deposition times. These results are well comparable with similar measurements reporter earlier by others (cf. Table S5). Some variability can be due to a dependence on slight preparation related variations of the lipid materials used, which have also been reported earlier (examples 1 and 3 in table S5, reported under the same conditions by the same authors, ~20% difference in D). D also depends strongly on the salt concentration (examples 8 and 9). Similar to our case, a higher DOTAP content has been associated with lower D, compare to POPC.

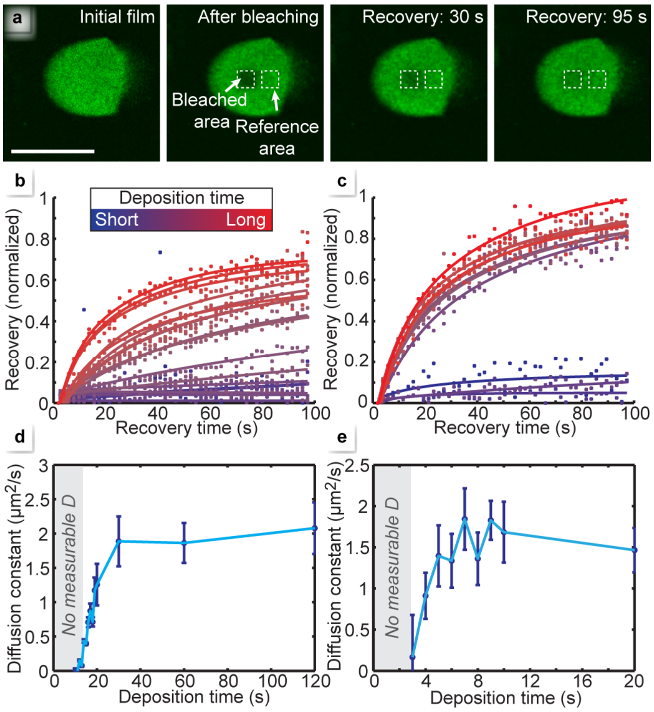

Fig S18. Results of the FRAP experiments, which represent an independent means to confirm the lipid film mobility. Exemplary image series of fluorescence recovery of a bleached POPC-488 film deposited for 20 s (**a**). Normalized experimental recovery curves for POPC-488 (**b**) and DOTAP-488 (**c**) films. The curves are coloured such, that the more blue (more red) colours are corresponding to shorter (longer) deposited films, respectively. The diffusion constant dependence on the deposition time is depicted in graphs (**d**) and (**e**), corresponding to POPC-488 and DOTAP-488, respectively. For short deposition times, where almost no recovery was observed, meaningful diffusion constants could not be extracted. The diffusion dependence on deposition exhibited a rather stepwise behaviour, meaning that a certain time is required before diffusive transport within the film is possible. However, after critical coverage is reached, no further increase in diffusion constant was observed even after prolonged deposition. These critical times were about 30 s for POPC and 6 s for DOTAP films, after which the diffusion constants remain unchanged at about ~ 2 μm^2/s and ~ 1.5 μm^2/s, and the mobile fractions ~72 % and ~100 % for POPC and DOTAP membranes, respectively.

Table S5. Comparison of transport properties for supported bilayers as reported previously. References are listed below the table.

Example	Lipid	Dye	Substrate	Environment	D	m	Ref.
**1**	egg PC	Rhodamine-DHPE	glass (piranha cleaned)	Tris/100mM NaCl	**2.79**	0.95	1
**2**	egg PC	35-nm dethered vesicles	glass (piranha cleaned)	Tris/100mM NaCl	**0.11**	0.86	1
**3**	egg PC	Rhodamine-DHPE	glass (piranha cleaned)	Tris/100mM NaCl	**3.3**		2
**4**	egg PC	Rhodamine-DHPE	glass	Tris/100mM NaCl	**3.18**	0.98	3
**5**	POPC	Rhodamine-DHPE	SiO_2 (CVD)	Tris/100mM NaCl (pH8)	**2**		4
**6**	POPC	Biotin-PE + SA-Cy3	glass	Tris/10mM NaCl (pH8)	**1.41**	0.93	5
**7**	POPC	NBD-eggPE	quartz	Tris/150mM NaCl (pH7.4)	**3.5**	0.8	6
**8**	POPC	TRITC-DHPE	quartz	100mM NaCl	**1.7**		7
**9**	POPC	TRITC-DHPE	quartz	pure water	**7.8**		7
**10**	DLPC	Rhodamine-DMPE	quartz	PBS (10mM)	**2.5**		8
**11**	POPC	Texas Red-DHPE	glass	PBS	**1.8**		9
**12**	POPC:DOTAP (3:7)	Texas Red-DHPE	glass	PBS	**1.2**		9
**13**	DOTAP	Texas Red-DHPE	glass	PBS	**0.6**		9
**14**	DOTAP	NBD C12-HPC	COC	pure water	**0.88**		10
**15**	POPC	NBD C12-HPC	COC	pure water	**2**		10

References

1 Jonsson, P., Jonsson, M. P., Tegenfeldt, J. O. & Hook, F. A Method Improving the Accuracy of Fluorescence Recovery after Photobleaching Analysis. *Biophysical Journal* **95**, 5334-5348, doi:10.1529/biophysj.108.134874 (2008).

2 Jonsson, P., Beech, J. P., Tegenfeldt, J. O. & Hook, F. Shear-Driven Motion of Supported Lipid Bilayers in Microfluidic Channels. *Journal of the American Chemical Society* **131**, 5294-5297, doi:10.1021/ja809987b (2009).

3 Jonsson, P., Beech, J. P., Tegenfeldt, J. O. & Hook, F. Mechanical Behavior of a Supported Lipid Bilayer under External Shear Forces. *Langmuir* **25**, 6279-6286, doi:10.1021/la8042268 (2009).

4 Jonsson, P., Jonsson, M. P. & Hook, F. Sealing of Submicrometer Wells by a Shear-Driven Lipid Bilayer. *Nano Letters* **10**, 1900-1906, doi:10.1021/nl100779k (2010).

5 Jonsson, P., Gunnarsson, A. & Hook, F. Accumulation and Separation of Membrane-Bound Proteins Using Hydrodynamic Forces. *Analytical Chemistry* **83**, 604-611, doi:10.1021/ac102979b (2011).

6 Kalb, E., Frey, S. & Tamm, L. K. FORMATION OF SUPPORTED PLANAR BILAYERS BY FUSION OF VESICLES TO SUPPORTED PHOSPHOLIPID MONOLAYERS. *Biochimica Et Biophysica Acta* **1103**, 307-316, doi:10.1016/0005-2736(92)90101-q (1992).

7 Bockmann, R. A., Hac, A., Heimburg, T. & Grubmuller, H. Effect of sodium chloride on a lipid bilayer. *Biophysical Journal* **85**, 1647-1655, doi:10.1016/s0006-3495(03)74594-9 (2003).

8 Zhang, L. F. & Granick, S. Lipid diffusion compared in outer and inner leaflets of planar supported bilayers. *Journal of Chemical Physics* **123**, doi:211104
10.1063/1.2138699 (2005).

9 Afanasenkau, D. & Offenhaeusser, A. Positively Charged Supported Lipid Bilayers as a Biomimetic Platform for Neuronal Cell Culture. *Langmuir* **28**, 13387-13394, doi:10.1021/la302500r (2012).

10 Hochrein, M. B., Reich, C., Krause, B., Radler, J. O. & Nickel, B. Structure and mobility of lipid membranes on a thermoplastic substrate. *Langmuir* **22**, 538-545, doi:10.1021/la051820y (2006).

S14. Membrane deposition dependence on operation parameters of the pipette

The following studies show, how the deposition process and spot geometry are depending on the operation parameters of the multifunctional pipette. Increase and decrease of the flow rates about 2 times had only a weak effect on the shape and deposition kinetics of the spot. On the other hand, we observed that changing the ouflow/inflow (OI) ratio changes the spot geometry from circular to elliptical (Figure S19) and reduces the achievable feature size quite considerably. Similarly, we studied the dependence on the height of the pipette (Figure S20). Both studies suggest that the deposition process is generally not very sensitive to small deviations in operation parameters, which indicated the overall robustness of liquid handing and dispensing of lipid material. The OI ratio dependence can potentially be utilized to purposefully achieve a reduced feature size of the deposits.

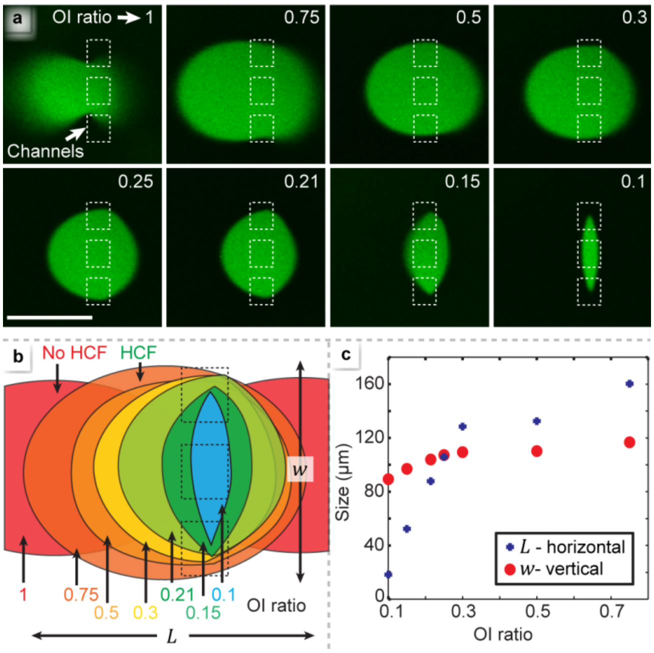

Fig S19. Deposition spot geometry depending on the outflow/inflow ratio (OI). The figure shows a series of micrographs of spots deposited at different OI (**a**). Schematically overlaid spot geometries (**b**), with dimensions depending on OI (**c**). OI is recommended to be 0.5 or less, to ensure contamination free operation. Initially the spot geometry is only weakly dependent on the OI, while later (OI<0.2) the spot is narrowing into an ellipse with its short axis perpendicular to the channel outlet array. The long axis of the elliptical spot (along the channel outlet array) is only weakly affected.

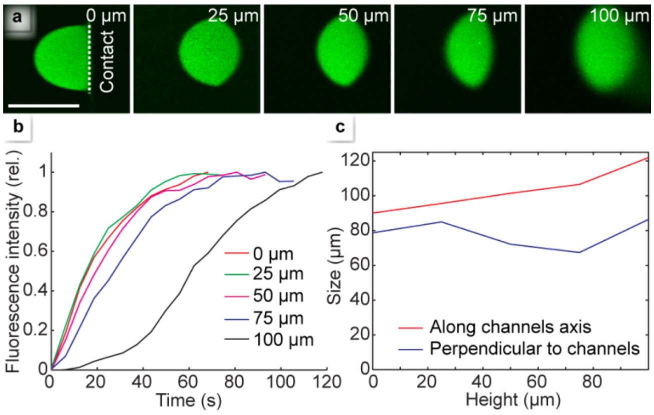

Fig S20. Deposition quality dependence on the pipette height from the surface. The series of micrographs shows lipid patches deposited at different pipette heights (**a**), a chart of the deposition kinetics for each height (**b**), and the spot geometry dependence on height (**c**). In all cases the pipette is operated in its regular mode (OI = 0.3). The series is starting from physical contact of the pipette tip with the substrate (0 µm) and continues in 25 µm steps to the final height of 100 µm. Vertical positioning has been carried out by a water-hydraulic micromanipulator. The deposition is initially only weakly dependent on the pipette height, which indicates good mechanical stability and robustness of the deposition process.

Supplementary Videos

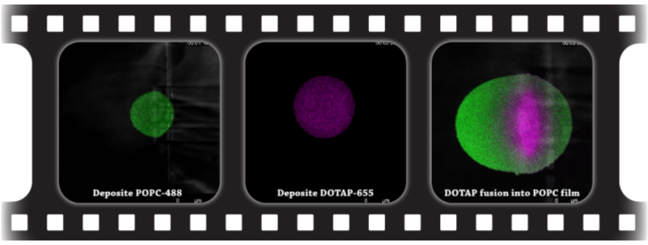

Video 1. Deposition. This is the supplementary video for figure 2 in the main text. This time-lapsed video shows deposition of POPC and DOTAP films, on-surface dilution of lipid, and fusion of DOTAP into a POPC film.

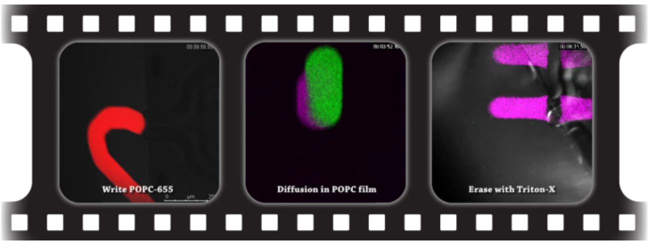

Video 2. Writing and erasing. This is the supplementary video for figure 3 in the main text. This time-lapsed video shows writing of single and multiple component membrane structures and 2D fluidic networks, further 2D diffusion in a membrane, and erasing and rewriting.

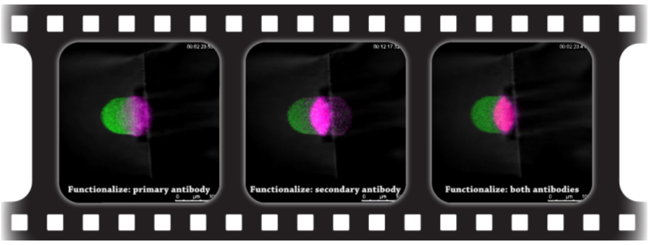

Video 3. Functionalization. This is the supplementary video for figure 4 in the main text. This time-lapsed video shows functionalization of membrane structures with a primary antibody (goat anti-biotin) and further functionalization with a secondary antibody (donkey anti-goat).

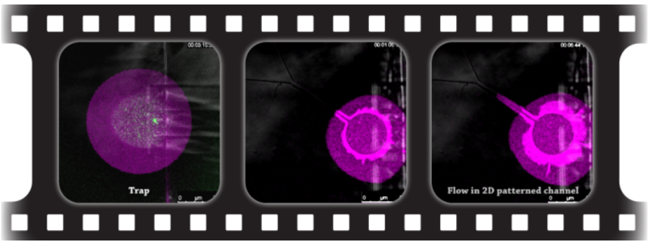

Video 4. Other examples. This is the supplementary video for figure 5 in the main text. This time-lapsed video shows hydrodynamic trapping of lipid vesicles from POPC adhered to a DOTAP film, and lipid flow on a surface patterned channel.
